# Weighted Road Density and Allergic Disease in Children at High Risk of Developing Asthma

**DOI:** 10.1371/journal.pone.0098978

**Published:** 2014-06-20

**Authors:** Anna L. Hansell, Nectarios Rose, Christine T. Cowie, Elena G. Belousova, Ioannis Bakolis, Kitty Ng, Brett G. Toelle, Guy B. Marks, for the Childhood Asthma Prevention Study Team, Childhood Asthma Prevention Study Team, plus Catarina Almqvist, Rosario D Ampon, Julian Ayer, Tessa Bird, Bronwyn K Brew, Warwick J Britton, David Celermajer, Christopher T Cowell, Daniele Crisafulli, Sally Criss, Stella Davis, Wafaa Nabil Ezz, Samantha Forbes, Frances L Garden, Andrew S Kemp, Natalia Knezevic, William Krause, Stephen R Leeder, Craig M Mellis, Seema Mihrshahi, Mark Neumann, Jennifer K Peat, Andres Quinones-Lucio, Michael Skilton, Anne Tattam, Euan R Tovey, Carl H. Vanlaar, Nicola Vukasin, Craig Wainwright, Karen L Webb, Christina Weber-Chrysochoou, Ann J Woolcock, Jie Zhou

**Affiliations:** 1 MRC-PHE Centre for Environment and Health, Imperial College London, London, United Kingdom; 2 Public Health and Primary Care Directorate, Imperial College Healthcare NHS Trust, London, United Kingdom; 3 Woolcock Institute of Medical Research, University of Sydney, Sydney, New South Wales, Australia; 4 New South Wales Health Ministry, Sydney, New South Wales, Australia; 5 South West Sydney Clinical School, University of New South Wales, Liverpool, New South Wales, Australia; 6 Sydney Local Health District, Sydney, New South Wales, Australia; 7 Department of Respiratory Medicine, Liverpool Hospital, Liverpool, New South Wales, Australia; Ludwig-Maximilians-University Munich, Germany

## Abstract

**Background:**

Evidence for an association between traffic-related air pollution and allergic disease is inconsistent, possibly because the adverse effects may be limited to susceptible subgroups and these have not been identified. This study examined children in the Childhood Asthma Prevention Study (CAPS), potentially susceptible to air pollution effects because of a family history of asthma.

**Methods:**

We examined cross-sectional associations at age eight years between road density within 75 m and 50 m of home address weighted by road type (traffic density), as a proxy for traffic-related air pollution, on the following allergic and respiratory outcomes: skin prick tests (SPTs), total and specific serum IgE, pre- and post-bronchodilator lung function, airway hyperresponsiveness, exhaled NO, and reported asthma and rhinitis.

**Results:**

Weighted road density was positively associated with allergic sensitisation and allergic rhinitis. Adjusted relative risk (RR) for house dust mite (HDM) positive SPT was 1.25 (95% CI: 1.06–1.48), for detectable house dust mite-specific IgE was 1.19 (95% CI: 1.01–1.41) and for allergic rhinitis was 1.30 (95% CI: 1.03–1.63) per 100 m local road or 33.3 m motorway within 50 m of home. Associations were also seen with small decrements of peak and mid-expiratory flows and increased risk of asthma, current wheeze and rhinitis in atopic children.

**Conclusion:**

Associations between road density and allergic disease were found in a potentially susceptible subgroup of children at high risk of developing atopy and asthma.

## Introduction

There have been conflicting findings from epidemiological studies examining the relationship between traffic-related air pollution and allergic disease in childhood [1]. The 2010 Health Effects Institute (HEI) review of traffic-related air pollution [2] concluded that evidence for a causal association between childhood asthma and living next to busy roads was between ‘sufficient’ and ‘suggestive but not sufficient’. However, it considered there was ‘inadequate and insufficient’ evidence to infer associations with IgE-mediated allergies, but noted that “…*the lack of consistency across epidemiology studies might have reflected a failure to identify susceptible subgroups.*” In contrast, toxicological and controlled human exposure studies have shown strong evidence for a relationship between diesel particle exposure and IgE-mediated allergic responses [2], [3]. For example, pre-exposure to diesel exhaust particles has been shown to enhance nasal sensitisation in humans [3]. Hence, there is currently a discrepancy between the state of epidemiological and the clinical and toxicological evidence on adverse health effects of traffic-related air pollution on allergic disease in children.

Possible reasons for the discrepancies among epidemiological studies include differences in measures of exposure (for example, different arrays of modelled or measured pollutants, proximity measures, traffic densities, and traffic counts), the methods of outcome assessment, and the characteristics of individuals studied [2]. Epidemiological, clinical and toxicological studies have suggested different phenotypes [4] of asthma and allergic rhinitis that may have different aetiologies. Atopy plays an important role in airway hyper-responsiveness [5] but self-reported asthma often occurs in the absence of atopy [6]. Self-reported asthma almost certainly represents a heterogeneous disease entity. Also, allergic disease manifestations and phenotypes may vary by age [4] and allergic diseases in childhood peak at different ages [7].

We hypothesised that children at high risk of developing allergic disease would be a susceptible subgroup with increased sensitivity to the effects of traffic-related air pollution. We used data derived from children in the Childhood Asthma Prevention Study (CAPS), a birth cohort of children born in Sydney, New South Wales (NSW) Australia where one or more parents or siblings had asthma or wheezing and included all children still living in the state of New South Wales. Original recruitment was to a randomised controlled trial of house dust mite (HDM) allergen avoidance and dietary fish oil supplementation [8] to age five years. Neither of the original interventions had an impact on prevalence of asthma-related outcomes at follow-ups to age eight years [9]. We examined associations between a marker of exposure to traffic-related air pollution and allergic disease, looking for consistency across different outcome measures. As complete address data and exposure data was only available at age 8 years, we conducted cross-sectional analyses to examine associations, using data for that point in time.

## Methods

Informed written consent was given by the parents of participating children and the study was approved by the Human Research Ethics Committees of the University of Sydney, Children's Hospital at Westmead, and by Sydney South West Area Health Services.

Nurse-administered questionnaires were used to obtain information on symptoms, diagnosed asthma and various environmental factors. Clinical assessment at age eight years included height, weight, allergen skin prick testing (SPT), blood samples for total and specific IgE, lung function and airway hyperresponsiveness testing and measurement of exhaled nitric oxide (eNO) [9]. A SPT was regarded as positive where the allergen weal was ≥3 mm. Serum specific IgE results were classified as negative (<0.35 kU_A_/L) or positive. Spirometric lung function (Forced Expiratory Volume in one second (FEV_1_), Forced Expiratory Flow at 50% Vital Capacity (FEF_50_), Forced Expiratory Flow at mid-expiratory phase (FEF_25–75_) and Forced Vital Capacity (FVC)) was measured pre- and post- administration of bronchodilator (salbutamol 200 µg). A methacholine challenge test was performed in all consenting children with baseline FEV_1_>70% predicted. Airway hyperresponsiveness (AHR) was defined as a fall in FEV_1_≥20% at or before administration of a cumulative dose ≤6.1 µmol methacholine. The measurement of bronchodilator response was conducted on a different day to all other clinical measurements. Please see File S1 for full details of clinical tests and questionnaire derived outcomes.

### Weighted road density exposure assignment

Weighted road density was used as an indicator of exposure to traffic-related air pollution using a model designed to predict air pollution for areas where air quality monitoring and traffic count data were not available [10]. Road density was represented by the weighted sum of the lengths of road within 75 m or 50 m radius of a 10×10 m grid property centroid, with motorways, arterial roads and primary roads given a weighting of 3, distributor roads a weighting of 2 and local roads given a weighting of 1. Radii of 75 m and 50 m were chosen given that concentrations of nitrogen dioxide (NO_2_), often used as a marker of traffic-related air pollution, have been shown to fall rapidly within that distance from roads [11];[12];[13]. Each study subject still residing in New South Wales was assigned the weighted road density score of the grid square in which the centroid of the address of their main place of residence at age eight years lay.

### Statistical analysis

Cross-sectional associations of weighted road density with binary allergic disease and sensitisation outcomes (SPTs, specific IgE, asthma, eczema and hayfever) were expressed as relative risks. Results were expressed per unit of weighted road density, where one unit relates to 100 m local road or 33.3 m of motorway within the given radius of the home. Due to problems with convergence in a log-binomial model, we used Poisson regression with robust error variance [14]. Multiple linear regression was used to analyse the effect of weighted road density on lung function (FEV_1_, FVC, FEV_1_ to FVC ratios), PEF, FEF_50_, FEF_25–75_, total IgE and eNO. Lung function analyses were conducted on log-transform variables and included covariates of gender, age at testing, weight and height.

Multivariate analyses adjusted for the following potential confounders identified *a priori*: gender, ethnicity, environmental tobacco exposure during pregnancy and childhood, breast-feeding to age 6 months, current or previous dog or cat ownership, gas heating, parental education. There was no *a priori* reason to associate either the original RCT dietary and HDM interventions with road traffic near home (and there were no significant differences in mean road density between the randomised groups) so these were not considered as confounders in our statistical analyses.

An *a priori* decision was made to conduct additional analyses for the lung function, AHR, eNO and questionnaire-reported diagnoses and symptoms stratifying by atopy (any positive SPT at age eight years) as individuals with atopy might be more sensitive to air pollution effects.

## Results

There were 616 children in the original birth cohort and for 560 of these had a most recent known address in New South Wales that could be geocoded. At age eight years there were 419 (75% of 560) children with questionnaire information on current asthma symptoms while 382 (68% of 560) children had results for skin prick tests (Table 1).

**Table 1:** Allergic sensitisation, self-reported allergic disease and lung function testing at age eight years.

Skin prick test >3 mm	Number (%)	n
Any of 11 inhalant and food allergens†	173 (45.3%)	382
Inhalant allergen‡	170 (44.5%)	382
Ingested allergen‡‡	30 (7.9%)	382
House dust mite (HDM)	137 (35.9%)	382
Ryegrass	69 (18.1%)	381
Grass mix	51 (13.4%)	380
Alternaria tenuis	43 (9.7%)	380
Cockroach	18 (4.7%)	381
Cat dander	23 (4.5%)	382
Aspergillus	14 (3.7%)	381
Dog	2 (0.5%)	381
**Total and specific IgE**		
Geometric mean total IgE Ku/L (geometric SD)	113.8 (5.1)	303
Any of the four specific IgEs measured ≥0.35 kUA/L	168 (52.7%)	319
HDM specific IgE≥0.35 kUA/L	138 (43.1%)	320
Ryegrass specific IgE≥0.35 kUA/L	91 (28.4%)	320
Alternaria tenuis specific IgE≥0.35 kUA/L	63 (19.8%)	319
Cat dander specific IgE≥0.35 kUA/L	29 (9.1%)	320
**Questionnaire reported atopic disease**		
Ever doctor-diagnosed asthma	170 (40.5%)	419
Wheeze in last 12 months	114 (27.2%)	419
Current asthma (wheeze+diagnosis/AHR)	93 (22.2%)	419
Current asthma (wheeze+asthma diagnosis)	91 (21.7%)	419
Cough in last 12 months	310 (74.0%)	419
Cough in last 12 months at least four times	101 (24.1%)	419
Poor asthma control at age 8 years	41 (9.8%)	419
Ever doctor diagnosed rhinitis	86 (20.5%)	419
Current rhinitis symptoms	110 (26.4%)	417
Ever doctor diagnosed eczema	204 (48.7%)	419
Current eczema	58 (14.3%)	405
**Exhaled NO**		
Geometric mean eNO ppb (geometric SD)	7.17 (1.98)	376

Spirometry, airways hyperresponsiveness	Mean (SD)	N
FEV_1_ pre bronchodilator % predicted	100.1% (11.8)	396
FVC pre bronchodilator % predicted	98.9% (11.6)	390
FEV_1_ post bronchodilator % predicted	105.9% (11.1)	392
FVC post bronchodilator % predicted	99.5% (10.6)	386
FEV_1_/FVC ratio pre bronchodilator	0.89 (0.07)	391
FEV_1_/FVC ratio post bronchodilator	0.93 (0.05)	387
Peak Expiratory Flow (PEF) pre bronchodilator	203(37.1)	410
Peak Expiratory Flow (PEF) post bronchodilator	211(38.2)	406
Forced expiratory flow at 50% vital capacity (FEF_50_) pre	2.18(0.55)	410
Forced expiratory flow at 50% vital capacity(FEF_50_) post	2.51(0.53)	406
Forced expiratory flow at mid-expiratory phase(FEF_25–75_) pre	2.11(0.43)	410
Forced expiratory flow at mid-expiratory phase(FEF_25–75_) post	2.35(0.43)	406
Airways hyperresponsiveness (AHR)	58 (17.5%)	332

† Any of egg white, egg yolk, salmon, tuna, peanuts, D. Pteronyssinus, cat dander, cockroach, alternaria, rye grass, grass mix, dog hair, aspergillus.

‡ Any of D. Pteronyssinus, cat dander, cockroach, rye grass, grass mix, alternaria, dog hair, aspergillus.

‡‡ Any of egg white, egg yolk, salmon, tuna, peanuts.

**Figure 1:**
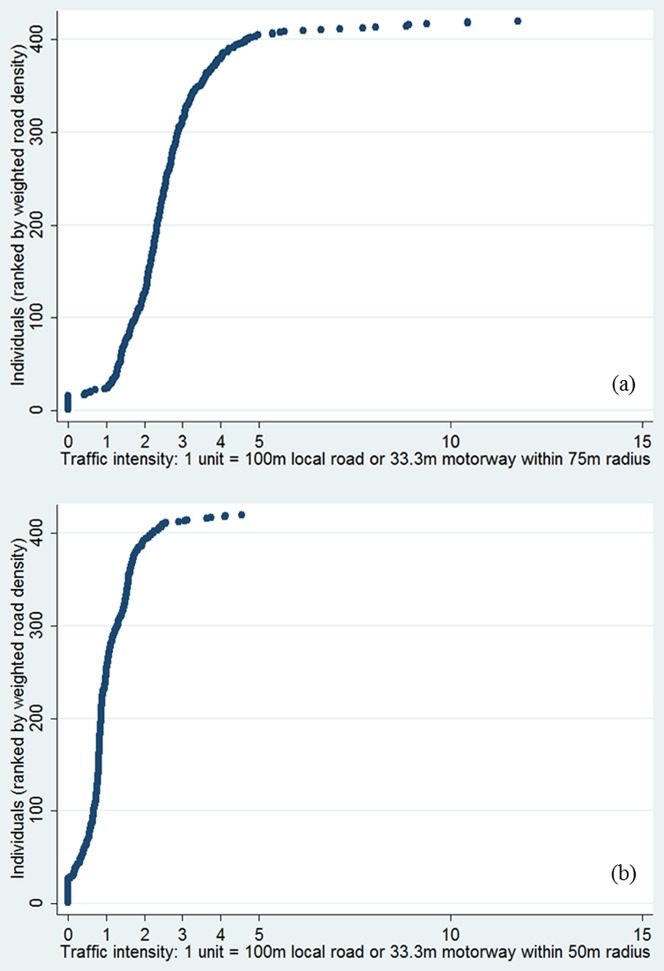
Traffic intensity distribution within 75(Figure 1a) and 50 m of home (Figure 1b) for all children with available questionnaire or clinical data, n = 419.

The distribution of the weighted road density variable is shown in Figure 1. For those present at the eight years follow-up, mean weighted road density within a 75 m radius of home was equivalent to 257 m of local road or 86 m motorway (median 240 m local road or 80 m motorway) and within a 50 m radius was equivalent to 103 m of local road or 34 m of motorway (median 88 m local road and 29 m motorway). The correlation between the two exposure variables was 0.7 (p<0.0001). Twenty-seven children had no roads within 50 m of the property centroid. There was no significant difference in weighted road densities for children without data at age eight years (Table S1 in File S1).

Forty-one percent of children had a recorded diagnosis of asthma at one or more assessments between 18 months and eight years and 22% had current asthma (Table 1). There was a high prevalence of allergen sensitisation, with 45% of children classified as atopic (positive skin prick test to any of 11 inhalant and food allergens). Forty-three percent of children had positive specific IgE levels to HDM; 82% of those with positive IgE levels also were SPT positive. Lung function tests showed mean values similar to those predicted. Fifty-eight children (17.5% of 332 tested) were classified as having airway hyperresponsiveness (AHR). Only two individuals were sensitised to dog hair on SPT, so this was omitted from regression analyses results.

Univariate analyses showed positive statistically significant associations with weighted road density within 50 m of home for inhaled allergens SPT and HDM SPT and HDM specific IgE (Table 2) and additionally with ryegrass and grass mix (SPT), cat dander (specific IgE) and alternaria (specific IgE) (Table S2 in File S1) and total IgE within 75 m of home (Table S3 in File S1). No significant univariate associations were seen with, eNO and questionnaire outcomes on the cohort as a whole except for doctor-diagnosed rhinitis (Table 3). Significant associations were also seen between weighted road density within 75 m of home and PEF, FEF_50_ and FEF_25–75_ (Table S3 in File S1) but not within 50 m, nor for other lung function measures.

**Table 2:** (Overall Sample) Univariate and multivariate logistic regression analysis for allergic sensitisation in relation to weighted road density within 50 m radius of home.

VARIABLES	N	RR	95% CI	N	RR	95% CI
	Unadjusted			Adjusted‡		
**Specific IgE**					**RR**	
House dust mite specific IgE> = 0.35 kU_A_/L	**320**	**1.21**	**1.03–1.42**	**311**	**1.19**	**1.01–1.41**
Ryegrass specific IgE> = 0.35 kU_A_/L	320	1.03	0.79–1.36	311	0.93	0.71–1.23
Alternaria specific IgE> = 0.35 kU_A_/L	319	1.19	0.89–1.59	310	1.07	0.78–1.46
Cat dander specific IgE> = 0.35 kU_A_/L	320	1.49	0.99–2.25	311	1.3	0.85–2.00
**Positive skin prick tests**						
Any	382	1.15	0.99–1.33	370	1.08	0.92–1.26
Inhalant allergens	**382**	**1.17**	**1.01–1.35**	370	1.1	0.95–1.29
Ingested allergens	382	1.1	0.72–1.69	370	0.95	0.58–1.57
House dust mite	**382**	**1.28**	**1.09–1.51**	**370**	**1.25**	**1.06–1.48**
Ryegrass	381	1.17	0.87–1.58	370	0.97	0.72–1.32
Grass mix	380	1.25	0.88–1.78	369	1.09	0.77–1.55
Alternaria	380	1.08	0.71–1.64	369	0.87	0.53–1.42
Cockroach	381	0.92	0.46–1.82	370	0.77	0.33–1.79
Cat dander	382	1.35	0.82–2.22	370	1.15	0.63–2.11
Aspergillus	381	0.69	0.32–1.51	370	0.69	0.27–1.76

Relative Risks (RRs) expressed per unit increase in weighted road density variable, where one unit relates to 100 m local road or 33.3 m of motorway within given radius of the home.

RR is the Relative Risk per unit increase in weighted road density from Poisson regression with robust standard errors conducted on binary variables.

‡ Multivariate analyses are adjusted for sex, father's education, mother's education, environmental tobacco smoke exposure, breastfed to 6 months, any dog owned by 8 years, any cat owned by 8 years, maternal smoking in pregnancy, gas cooking at home.

**Table 3:** (Overall Sample) Univariate and multivariate linear regression for spirometry and total IgE (kU/L) and Poisson regression with robust standard error analyses for AHR, eNO, questionnaire outcomes (all) in relation to weighted road density within 50 m radius of home.

Variables		Unadjusted				Adjusted‡		
	N	Percent difference (B)	95% CI	p-value interaction term with atopy‡‡	N	Percent difference (B)	95% CI	p-value interaction term with atopy‡‡
**Total IgE**								
Total IgE (kU/L) †	303	27.39	−6.40–73.38	-	294	25.41	−9.41–73.60	**-**
**Spirometry**								
FEV1 pre bronchodilator (L)	397	−0.66	−2.37–1.07	0.914	392	−0.59	−2.35–1.20	0.515
FEV1 post bronchodilator (L)	393	−0.31	−1.84–1.25	0.456	388	−0.35	−1.92–1.25	0.666
FVC pre bronchodilator (L)	391	−0.2	−1.90–1.52	0.7	386	−0.08	−1.83–1.70	0.929
FVC post bronchodilator (L)	387	−0.55	−2.12–1.04	0.545	382	−0.46	−2.06–1.15	0.571
FEV1/FVC ratio pre bronchodilator	391	−0.54	−1.67–0.61	0.979	386	−0.61	−1.79–0.59	0.316
FEV1/FVC ratio post bronchodilator	387	0.17	−0.68–1.02	0.634	382	−0.01	−0.88–0.87	0.99
pre Peak Expiratory Flow (PEF)	268	−1.14	−4.68–2.54	0.429	265	−2.09	−5.81–1.78	0.284
post Peak Expiratory Flow (PEF)	264	−1.78	−5.21–1.77	0.444	261	−2.28	−5.86–1.45	0.226
Pre forced expiratory flow at 50% vital capacity (FEF_50_)	268	−0.19	−5.09–4.97	0.567	265	−2.02	−7.03–3.26	0.445
Post forced expiratory flow at 50% vital capacity(FEF_50_)	264	−2.84	−6.72–1.21	0.271	261	−4.11	−8.06–0.01	0.0504
Pre forced expiratory flow at mid-expiratory phase(FEF_25–75_)	268	−0.95	−4.93–3.19	0.48	265	−2.33	−6.45–1.97	0.282
Post forced expiratory flow at mid-expiratory phase(FEF_25–75_)	264	−2.27	−5.75–1.34	0.393	261	−3.06	−6.63–0.65	0.105
		**RR**				**RR**		
eNO ppb †	376	1.02	0.96–1.06	0.94	364	1.04	0.97–1.07	0.923
AHR (yes/no)	332	0.96	0.62–1.48	**0.0105**	321	0.95	0.63–1.41	**0.0075**
**Questionnaire outcomes**		**RR**				**RR**		
Ever doctor-diagnosed asthma	419	1.09	0.92–1.28	0.201	398	1.04	0.87–1.23	0.265
Wheeze in last 12 months	419	1.19	0.96–1.47	0.24	398	1.12	0.90–1.39	0.251
Cough in last 12 months	419	1.03	0.94–1.12	0.311	398	1.02	0.94–1.12	0.346
Cough more than 4 times in last 12 months	419	1.19	0.94–1.50	0.226	398	1.18	0.93–1.49	0.226
Current asthma (wheeze+diagnosis/AHR)	419	1.2	0.94–1.54	0.469	398	1.12	0.87–1.45	0.546
Current asthma (wheeze+asthma diagnosis)	419	1.22	0.95–1.56	0.485	398	1.13	0.87–1.46	0.539
Poor asthma control	419	1.3	0.87–1.95	0.885	398	1.32	0.92–1.89	0.971
Ever doctor-diagnosed rhinitis	**419**	**1.37**	**1.11–1.69**	0.897	**398**	**1.30**	**1.03–1.63**	0.825
Current rhinitis symptoms	417	1.21	0.97–1.50	0.0856	397	1.18	0.95–1.47	0.156
Ever doctor-diagnosed eczema	419	1.11	0.96–1.27	0.565	398	1.11	0.96–1.28	0.424
Current eczema	405	1.30	0.98–1.74	0.358	392	1.17	0.86–1.61	0.333

Relative Risks (RRs) and percent difference (B) expressed per unit increase in weighted road density variable, where one unit relates to 100 m local road or 33.3 m of motorway within given radius of the home.

Lung function variables are logged, therefore percent differences by B = 100(e^β^−1) are presented for each unit increase in weighted road density, where β is the regression coefficient from linear regression analyses for continuous variables.

RR is the Relative Risk per unit increase in weighted road density from Poisson regression with robust standard errors conducted on binary variables.

‡ Multivariate analyses are adjusted for sex, father's education, mother's education, environmental tobacco smoke exposure, breastfed to 6 months, any dog owned by 8 years, any cat owned by 8 years, maternal smoking in pregnancy, gas cooking at home. Univariate and multivariate lung function analyses included adjustment for age, height and weight.

‡‡ p-value from additional analysis where an interaction term of weighted road density and atopy was included in the model.

**Figure 2:**
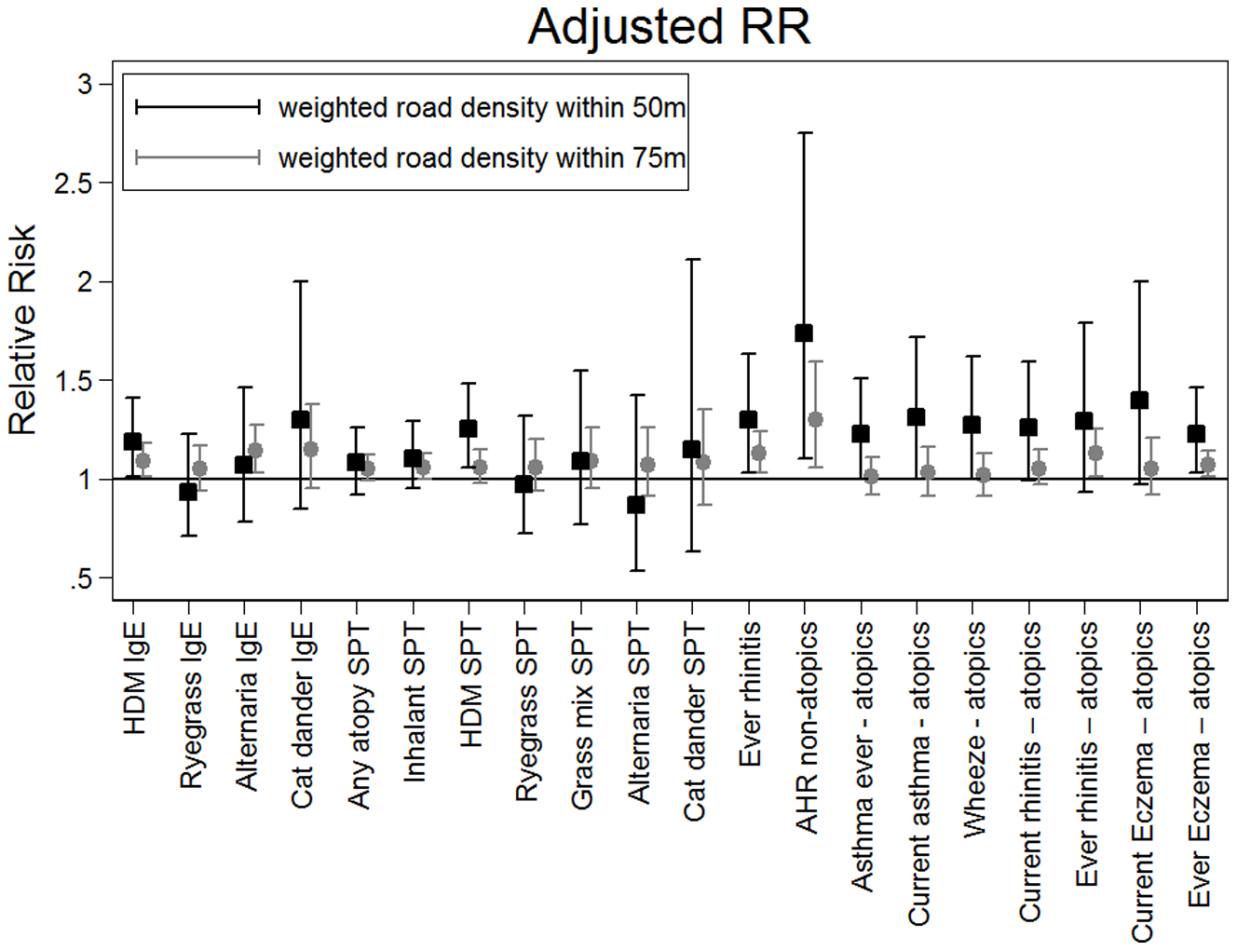
Relative risks and 95% confidence intervals from multivariate analysis of allergic sensitisation in relation to weighted road density within 75 m radius and 50 m radius of home. RRs expressed per unit increase in weighted road density variable, where one unit relates to 100's education, mother's education, environmental tobacco smoke exposure, breastfed to 6 months, any dog owned by 8 years, any cat owned by 8 years, maternal smoking in pregnancy, gas cooking at home.

**Figure 3:**
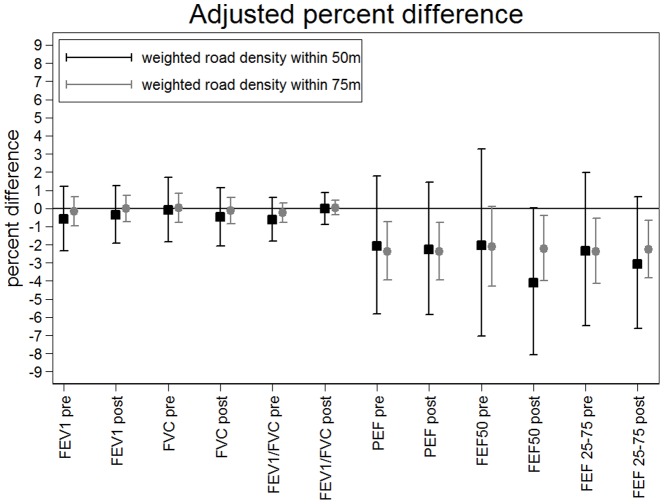
Percent difference and 95% confidence intervals from multivariate analysis of lung function and flow variables in relation to weighted road density within 75 m radius and 50 m radius of home. Percent difference expressed per unit increase in weighted road density variable, where one unit relates to 100

In multivariate analyses (Table 2 & 3, Figure 2) the strongest associations were seen for allergic rhinitis and HDM sensitisation for weighted road density within 50 m of home. There was a 25% increase in risk of a positive skin prick test to HDM per unit increase of weighted road density within 50 m of home (RR 1.25, 95% CI: 1.06–1.48) and a corresponding 19% increased risk of raised HDM specific IgE (RR 1.19, 95% CI: 1.01–1.41) where one unit represents 100 m local road or 33.3 m motorway or equivalent. There was also a statistically significant increase in Total IgE and alternaria specific IgE for weighted road density within 75 m of home, but not for 50 m where confidence intervals were wider (Figure 2, Table S2 in File S1). For doctor-diagnosed allergic rhinitis, the RR for all individuals was 1.30 (95% CI: 1.03–1.63). RRs were generally (although not always) slightly higher for traffic density within 50 m than 75 m (Figure 2). For lung function, significant multivariate associations were seen suggesting small decreases of pre-and post bronchodilator PEF, FEF_50_ and FEF_25–75_ in association with weighted road density within 75 m but this did not reach statistical significance for weighted road density within 50 m (Figure 3, Table 2, Table S3 in File S1).

Interaction terms for atopy status were only statistically significant for AHR (see interaction term p-values Table 3 and Table S3 in File S1). However, in multivariate stratified analyses (Tables S4–S7 in File S1, Figure 2), statistically significant associations with weighted road density were seen in atopics with current and ever doctor-diagnosed asthma, with wheeze, rhinitis and ever doctor-diagnosed eczema and (for weighted road density within 75 m of home) with PEF, FEF_50_, FEF_25–75_ (Tables S4 & S6 in File S1), as well as for AHR in non-atopic subjects (Tables S5 & S7 in File S1).

## Discussion

This study found cross-sectional associations between a measure of traffic intensity and allergic sensitisation and allergic rhinitis in children aged eight years, potentially at increased risk of sensitisation because of a family history of asthma. Traffic intensity was also associated with small decrements in mid and peak expiratory flow (but not other lung function measures) and with current asthma, current wheeze and rhinitis in atopic children and with AHR in non-atopic children.

The prevalence of current asthma (22%) and AHR (17.5%) was slightly higher than in unselected populations in Australia, probably explained by the high risk nature of the cohort [15]. The children had a higher prevalence of sensitisation to inhalant allergens (43% positive for HDM specific IgE) than European population-based cohorts of similar age [16];[17], but similar atopy prevalence to other children of this age in Australia [18].

Lack of information on measured or modelled air pollution concentrations is a limitation of the study and makes direct comparisons with published studies that use these measures difficult. However, assessment of coherence across different study designs can be made. As air pollution estimates at place of residence were not available, weighted road density was used as an indicator of traffic exposure. Weighted road density was found to be as strongly related to NO_2_ as traffic volumes in a previous study in 2006–7 using passive samplers at 38 monitoring sites in Sydney [10]. However, using data on exposures to specific air pollution components would have been informative as different components may have different mechanisms. Some studies have suggested that a combination of particulates and allergens can enhance induction of allergic disease and/or allergic responses but that NO_2_ enhances reactions to allergens only in those with pre-existing susceptibility (for example, asthma) [2], presumably due to its irritant properties. As in many air pollution studies, exposure at home address was used as a proxy for exposure as we did not have information on school location or person-time movements. This simplifying assumption may have introduced some exposure misclassification, but this is most likely to be random and have resulted in bias towards the null.

A further limitation of this report is that it is a *post hoc* evaluation, addressing a different question to that posed at the inception of the birth cohort and that, although consistent with findings from toxicological studies in terms of relationships between diesel particle exposure and allergic responses [2], the study's cross-sectional design precludes conclusions with respect to cause and effect. Additionally, we conducted a large number of statistical tests, so some significant associations may be chance findings and therefore would need confirmation by replication in other studies.

RRs were generally higher for traffic density within 50 m than 75 m (Figure 2), which is consistent with the very steep decline in concentration of many traffic-related air pollutants within the first 100 m distance from roads [2], [11]. In previous studies, the strongest associations with allergic disease, such as asthma, eczema and hayfever, and with allergic sensitisation have been seen within 50 m [17] to 100 m [19] of a road with a reduction in effect size with increasing distance [17], [20]. Studies examining exposures within 150 m from a major road [21] or local road traffic activity in 1 km grid squares [22], [23] have found inconsistent or no effects on wheeze and allergic disease.

Our findings on allergic sensitisation are consistent with those in two German cohorts of comparable age [17], where significant associations were found between specific IgE to inhalant allergens measured at age six years and both PM_2.5_ and distance to road. In the Dutch PIAMA cohort [16] positive but non-significant associations were seen between specific IgEs measured at age eight years and NO_2_, PM_2.5_ or when assessed at age two-three years; significant associations were previously found in this cohort at age four years [24] when a higher proportion of those tested had allergic mothers than at age eight years (67% at age four years vs. 38% at age eight years). The Swedish BAMSE cohort [25] found an effect on allergic sensitisation at age 8 years of modelled traffic emissions for NOx and road dust related PM_10_ at age 0–1 years but not age 1–4 or 4–8 years. However, the European Study of Cohorts for Air Pollution Effects (ESCAPE) meta-analysis with five cohorts, including the four mentioned previously, did not find overall significant associations between air pollution and sensitisation (based on specific IgE) at either 4–6 or 8–10 years [26]. Unlike these European cohorts [16], [17], [25], the strongest effects here were seen with HDM sensitisation. Sydney has a warm climate and it is usual to leave windows and doors open other than in winter. The potential mechanism for dust mite sensitisation with respect to traffic pollutants is therefore likely to be the same as for pollen.

Traffic-related pollutant exposures were lower in New South Wales (NSW) than in European [27], [28] Asian [29] or American studies [30]–[32]. Annual average NO_2_ levels in Sydney and NSW were 15 ppb or less in 2000–2008 [10], [33], lower than the mean levels of many of the areas in these other studies.

Several cross-sectional and cohort studies have reported deficits in lung function in children associated with traffic-related air pollution [28], [34]–[38], while others have not or found only weak associations [23], [39], [40]. Within the ESCAPE analysis of five European cohorts [34], small negative associations were seen between PEF and NO_2_ at current address but not traffic load on major roads within 100 m buffer. The lower levels of air pollution may partly explain why we did not find significant associations of traffic intensity with FEV_1_ and FVC, unlike some other studies of children of similar ages but with higher levels of air pollution [34], [41]. However, we did see associations between the traffic intensity measures and small decrements of pre- and post-bronchilator PEF, FEF_50_ and FEF_25–75_ (although only statistically significant for weighted road density with 75 m but not 50 m of home). Other explanations for our inconsistent findings include exposure misclassification bias and limited statistical power.

Few studies have examined whether atopic status may affect sensitivity to air pollution. In the present study, significant associations were seen between weighted road density and allergic rhinitis, asthma and wheeze in atopic children but not in non-atopic children and with AHR in non-atopic children. An analysis reported from the BAMSE cohort [41] suggested effects on lung function in atopic children, while the PIAMA cohort suggested effects of air pollutants on asthma in non-atopic but not in atopic children [16].

Socioeconomic status may be an important confounder in studies of air pollution and asthma. Parental education was adjusted for in the models. Exposures (mean road densities) were not statistically different between levels of parental education (not shown), but a higher percentage of children with missing clinical or questionnaire data were from families with lower parental educational levels (Table S1 in File S1), which is a potential source of bias.

This study showed associations between exposure to weighted road density and the prevalence of allergic sensitisation and potentially also small airway function in children with a family history of asthma. Although the cross-sectional design and the indirect method of exposure estimation limit the causal inferences that can be drawn, it does provide some epidemiological support for toxicological studies that have demonstrated a relationship between diesel particle exposure and IgE-mediated allergic responses [2], [3]. Study of birth cohorts with prospectively collected data on exposure to traffic-related air pollutants are warranted to further elucidate the role of this exposure on the risk of developing atopy and allergic disease.

## Supporting Information

File S1 **Supplementary methods and Tables S1–S7. Table S1.** Comparison of weighted road density exposure and of major confounders for those with missing and non-missing data. **Table S2.** (Overall Sample). Univariate and multivariate logistic regression analysis for allergic sensitisation in relation to weighted road density within 75 m radius of home. Relative Risks (RRs) expressed per unit increase in weighted road density variable, where one unit relates to 100 m local road or 33.3 m of motorway within given radius of the home. **Table S3.** Overall Sample in relation to weighted road density within 75 m radius of home. Univariate and multivariate linear regression for spirometry and Poisson regression with robust standard error analyses for AHR, eNO, questionnaire outcomes (all) in relation to weighted road density within 75 m radius of home. **Table S4.** Atopics in relation to weighted road density within 50 m radius of home. Univariate and multivariate linear regression for spirometry and Poisson regression with robust standard error analyses for AHR, eNO, questionnaire outcomes (all) in relation to weighted road density within 50 m radius of home. **Table S5.** Non –atopics in relation to weighted road density within 50 m radius of home. Univariate and multivariate linear regression for spirometry and Poisson regression with robust standard error analyses for AHR, eNO, questionnaire outcomes (all) in relation to weighted road density. **Table S6.** Atopics in relation to weighted road density within 75 m radius of home. Univariate and multivariate linear regression for spirometry and Poisson regression with robust standard error analyses for AHR, eNO, questionnaire outcomes (all) in relation to weighted road density within 75 m radius of home. **Table S7.** Non-Atopics in relation to weighted road density within 75 m radius of home. Univariate and multivariate linear regression for spirometry and Poisson regression with robust standard error analyses for AHR, eNO, questionnaire outcomes (all) in relation to weighted road density within 75 m radius of home.
(DOCX)
