# Supplementary material for: Weighted Road Density and Allergic Disease in Children at High Risk of Developing Asthma
Source: PLoS One. 2014 Jun 20;9(6):e98978. doi: 10.1371/journal.pone.0098978 (PMC4064977; doi:10.1371/journal.pone.0098978)
Supplement: File S1 — Supplementary methods and Tables S1–S7. Table S1. Comparison of weighted road density exposure and of major confounders for those with missing and non-missing data. Table S2. (Overall Sample). Univariate and multivariate logistic regression analysis for allergic sensitisation in relation to weighted road density within 75 m radius of home. Relative Risks (RRs) expressed per unit increase in weighted road density variable, where one unit relates to 100 m local road or 33.3 m of motorway within given radius of the home. Table S3. Overall Sample in relation to weighted road density within 75 m radius of home. Univariate and multivariate linear regression for spirometry and Poisson regression with robust standard error analyses for AHR, eNO, questionnaire outcomes (all) in relation to weighted road density within 75 m radius of home. Table S4. Atopics in relation to weighted road density within 50 m radius of home. Univariate and multivariate linear regression for spirometry and Poisson regression with robust standard error analyses for AHR, eNO, questionnaire outcomes (all) in relation to weighted road density within 50 m radius of home. Table S5. Non –atopics in relation to weighted road density within 50 m radius of home. Univariate and multivariate linear regression for spirometry and Poisson regression with robust standard error analyses for AHR, eNO, questionnaire outcomes (all) in relation to weighted road density. Table S6. Atopics in relation to weighted road density within 75 m radius of home. Univariate and multivariate linear regression for spirometry and Poisson regression with robust standard error analyses for AHR, eNO, questionnaire outcomes (all) in relation to weighted road density within 75 m radius of home. Table S7. Non-Atopics in relation to weighted road density within 75 m radius of home. Univariate and multivariate linear regression for spirometry and Poisson regression with robust standard error analyses for AHR, eNO, questionnaire outcomes (al [file pone.0098978.s001.docx]

**Traffic density and allergic disease in children age eight years at high risk of developing asthma - Online Supplement**

Contents

[Supplementary Methods 2](#_Toc384406084)

[Questionnaires 2](#_Toc384406085)

[Clinical data 3](#_Toc384406086)

[Road density exposure assignment 5](#_Toc384406087)

[Additional information on statistical analysis 6](#_Toc384406088)

[Reference list for supplementary methods 6](#_Toc384406089)

[Table S1 Comparison of weighted road density exposure and of major confounders for those with missing and non-missing data 7](#_Toc384406090)

[Table S2 (Overall Sample). Univariate and multivariate logistic regression analysis for allergic sensitisation in relation to weighted road density within 75m radius of home. Relative Risks (RRs) expressed per unit increase in weighted road density variable, where one unit relates to 100m local road or 33.3m of motorway within given radius of the home 8](#_Toc384406091)

[Table S3 Overall Sample in relation to weighted road density within 75m radius of home . Univariate and multivariate linear regression for spirometry and Poisson regression with robust standard error analyses for AHR, eNO, questionnaire outcomes (all) in relation to weighted road density within 75m radius of home 9](#_Toc384406092)

[Table S4 Atopics in relation to weighted road density within 50m radius of home. Univariate and multivariate linear regression for spirometry and Poisson regression with robust standard error analyses for AHR, eNO, questionnaire outcomes (all) in relation to weighted road density within 50m radius of home. 11](#_Toc384406093)

[Table S5 Non –atopics in relation to weighted road density within 50m radius of home. Univariate and multivariate linear regression for spirometry and Poisson regression with robust standard error analyses for AHR, eNO, questionnaire outcomes (all) in relation to weighted road density 13](#_Toc384406094)

[Table S6 Atopics in relation to weighted road density within 75m radius of home. Univariate and multivariate linear regression for spirometry and Poisson regression with robust standard error analyses for AHR, eNO, questionnaire outcomes (all) in relation to weighted road density within 75m radius of home 15](#_Toc384406095)

[Table S7 Non-Atopics in relation to weighted road density within 75m radius of home. Univariate and multivariate linear regression for spirometry and Poisson regression with robust standard error analyses for AHR, eNO, questionnaire outcomes (all) in relation to weighted road density within 75m radius of home 17](#_Toc384406096)

## Supplementary Methods

### Questionnaires

Nurse-administered questionnaires were used to obtain information on symptoms, diagnosed asthma and various environmental factors. Information about smoking in pregnancy was obtained by interview soon after delivery of the child. Additionally, study nurses collected data on environmental tobacco smoke exposure at regular home visits and telephone calls during pregnancy up to age 7 ½ years.

For analyses, “asthma ever” was defined as a doctor diagnosis of asthma reported in at least one follow-up (at 18 months, 3, 5 or 8 years). Current asthma was defined as wheeze in the last 12 months plus "asthma ever" ("Current asthma (wheeze + asthma diagnosis)") or as wheeze in the last 12 months plus “asthma ever" or the presence of AHR ("Current asthma (wheeze + diagnosis/AHR)"). Ever rhinitis was defined as a doctor diagnosis of allergic rhinitis at one or more assessment. Current rhinitis was defined as sneezing, rhinorrhoea or blocked nose without a cold. Eczema was defined as either eczema on examination at age 8 years or a reported recurrent itchy rash together with use of topical treatments for eczema in the last 12 months. Poor asthma control at age eight years was defined as the presence of one or more of the following: 12 or more episodes of wheeze per year, disturbed sleep one or more nights per week, using bronchodilator for night or early morning symptoms three or more times per week, and visiting a doctor, Emergency Department or hospital for wheeze in the last 12 months.

The questionnaire was developed from a number of sources. Firstly, we used questions that had been used during previous CAPS assessments to allow for consistency of definitions between follow-ups [1]. Secondly, we added in validated questions from the International Study of Asthma and Allergy (ISAAC) 6-7 year old core questionnaire to allow for comparison between this cohort and other international cohorts [2]. Finally, we designed questions that were consistent with the Third International Paediatric Consensus Statement on the Management of Childhood Asthma to allow classification of asthma to be consistent with the current consensus statement [3].

### Clinical data

Clinical assessment at age eight years included height, weight, allergen skin prick testing (SPT), blood samples for total and specific IgE, lung function, airway hyper-responsiveness testing and measurement of exhaled nitric oxide (eNO) [4].

Atopy was measured using SPT as previously described [1]. Glycerol and histamine phosphate (10mg/ml) were used as negative and positive controls, respectively. Food allergens tested were salmon, peanut mix, egg white, egg yolk, and tuna, and inhalant allergens tested were house dust mite *Dermatophagoides pteronyssinus* (HDM), cockroach, cat pelt, dog hair, *Alternaria alternata (tenuis)*, *Aspergillus fumigatus*, rye grass and a grass mix (Hollister-Stier, Spokane WA). Weals were measured at 10 minutes as the mean of the longest diameter and its perpendicular; values were rounded down to the nearest 1mm. A positive test was one where the weal was ≥3mm and also greater than the negative control weal.

In addition to total serum IgE, specific IgE was measured for HDM, cat dander, Alternaria and rye grass using the ImmunoCAP procedure (Pharmacia CAP System, Pharmacia Diagnostics AB, Uppsala, Sweden). Serum specific IgE results were classified as negative (< 0.35 kU_A_/L) or positive.

Lung function (Forced Expiratory Volume in one second (FEV1) and Forced Vital Capacity (FVC)) was measured using a Spirocard (QRS Diagnostic, LLC, Plymouth, MN, USA) spirometer linked to a laptop computer running SpiroScore+ (V2.6) software (Bird Healthcare, Melbourne, Australia). Children also had reversibility measured 10 minutes after the administration of salbutamol (200 µg) via a large volume spacer. The measurement of bronchodilator response was conducted on a different day to all other clinical measurements. Predicted lung function values were calculated using NHANES III equations [5].

Airway hyperresponsiveness (AHR) was measured using a methacholine inhalation challenge test according to the rapid method[6]. Children were defined as having AHR if the FEV_1_ fell by 20% or more from the post-saline value (PD_20_FEV_1_ was < 6.1 μmol methacholine), if they had deep inspiration (DI)-induced bronchoconstriction (FEV_1_ after normal saline was >10% below the first baseline FEV_1_ (two children) and those who had a > 15% bronchodilator response on spirometric testing but did not complete a methacholine challenge for various reasons (refusal, inability to perform, or low baseline FEV_1_ (<70%) –total of six children).

Our spirometry protocol was consistent with ATS/ERS lung function testing criteria [7]. Post-bronchodilator spirometry was performed by a single experienced research nurse trained in spirometry. Airway hyperresponsiveness challenge and accompanying spirometry was performed by three experienced research staff trained in spirometry. One single spirometer was used for all measurements. The spirometer was calibrated each day and was re-checked mid-way through the day for an extended day of testing. Selection of best value was according to ATS guidelines criteria for useable and acceptable curves that was programmed into the spirometry software.

Exhaled nitric oxide (eNO) was measured at age eight years using the offline technique [8] with subject standing and with a nose clip on. The subject inhaled and exhaled five breaths of room air through a scrubber to remove NO and then exhaled through a rotameter at about 200 mls per second (by keeping a floating ball between two sets of flow rate markers). The first one to two seconds of expiration were discarded to exclude dead space air, then airflow was directed into a 3L impermeable polyethylene (Scholle Industries, Adelaide, Australia) collecting bag. The subject was instructed to continue to exhale down to near residual volume. This procedure was repeated two or three times until approximately 1L was collected into the bag. Within 24 hours of collection (usually same day), the contents were aspirated into a chemiluminescent analyser (Model 42C; Thermo Environmental Instruments, Franklin, MA) to measure nitric oxide (NO) concentration in parts per billion (ppb).

### Road density exposure assignment

Road density, as a measure of traffic related air pollution, was assigned to the main place of residence for the child. Addresses for the cohort have been stored electronically from age eight and used for follow-up purposes. Addresses were geocoded to the centroid of the property parcel (address block) using FEBRL (Freely Extensible Bio-medical Record Linkage) software developed by NSW Health and the Australian National University [9]. This study was restricted to children living in Sydney or elsewhere within the state of New South Wales at the age of eight years.

### Additional information on statistical analysis

Adjusted RRs for cat specific IgE were computed omitting cat ownership as a covariate as this may be on the causal pathway for sensitisation, but RRs were nearly identical (data not shown).

### Reference list for supplementary methods

^1. Peat JK, Mihrshahi S, Kemp AS, Marks GB, Tovey ER, Webb K, Mellis CM, Leeder SR (2004) Three-year outcomes of dietary fatty acid modification and house dust mite reduction in the Childhood Asthma Prevention Study. J Allergy Clin Immunol 114: 807-813.^

^2. Asher MI, Keil U, Anderson HR, Beasley R, Crane J, Martinez F, Mitchell EA, Pearce N, Sibbald B, Stewart AW, Strachan D, Weiland SK, Williams HC (1995) International Study of Asthma and Allergies in Childhood (ISAAC): rationale and methods. Eur Respir J 8: 483-491.^

^3. Warner JO, Naspitz CK, Cropp GJA (1998) Third International Pediatric Consensus statement on the management of childhood asthma. Pediatr Pulmonol 25: 1-17.^

^4. Toelle BG, Ng KK, Crisafulli D, Belousova EG, Almqvist C, Webb K, Tovey ER, Kemp AS, Mellis CM, Leeder SR, Marks GB (2010) Eight-year outcomes of the Childhood Asthma Prevention Study. J Allergy Clin Immunol 126: 388-389.^

^5. Hankinson J, Odencrantz J, Fedan K (1999) Spirometric Reference Values from a Sample of the General U.S. Population. Am J Respir Crit Care Med 159: 179-187.^

^6. Yan K, Salome C, Woolcock AJ (1983) Rapid method for measurement of bronchial responsiveness. Thorax 38: 760-765.^

^7. Miller MR, Hankinson J, Brusasco V, Burgos F, Casaburi R, Coates A, Crapo R, Enright P, van der Grinten CPM, Gustafsson P, Jensen R, Johnson DC, MacIntyre N, McKay R, Navajas D, Pedersen OF, Pellegrino R, Viegi G, Wanger J (2005) Standardisation of spirometry. Eur Respir J 26: 319-338.^

^8. Salome CM, Roberts AM, Brown NJ, Dermand J, Marks GB, Woolcock AJ (1999) Exhaled nitric oxide measurements in a population sample of young adults. Am J Respir Crit Care Med 159: 911-916.^

^9. Summerhayes R, Holder P, Beard J, Morgan G, Christen P, Willmore A, Churches T (2006) Automated geocoding of routinely collected health data in New South Wales. N S W Public Health Bull 17: 33-38.^

## Table S1 Comparison of weighted road density exposure and of major confounders for those with missing and non-missing data

|  | **With some clinical or questionnaire data age 8 years**  **(N=419)** | **No clinical or questionnaire data age 8 years**  **(N=141)** | **p-value**† |
| --- | --- | --- | --- |
|  |  |  |  |
| Weighted road density within 75m of home (m) | 256 | 276 | 0.201 |
| Weighted road density within 50m of home (m) | 104 | 102 | 0.833 |
| Father’s education (% below university-level) | **52.3%** | **68.6%** | **0.008** |
| Mother’s education (% below university level) | **50.6%** | **70.9%** | **<0.001** |
| Male | 51.8% | 51.8% | 0.997 |
| Breast-feeding for six months | 42.0% | 44.7% | 0.578 |
| Dog ownership | 63.7% | 59.1% | 0.341 |
| Cat ownership | 18.9% | 15.0% | 0.317 |
| Mother smoking during pregnancy | 23.2% | 27.0% | 0.362 |

Results for environmental tobacco smoke and gas cooking not shown as missing data on these for ~100 children make comparisons unreliable.

† p-values from chi-sq test or t-test for weighted road density. (For education, p-value tests four levels (primary, high school year 10, high school year 12, university or equivalent)

## Table S2 (Overall Sample). Univariate and multivariate logistic regression analysis for allergic sensitisation in relation to weighted road density within 75m radius of home. Relative Risks (RRs) expressed per unit increase in weighted road density variable, where one unit relates to 100m local road or 33.3m of motorway within given radius of the home

| **VARIABLES** | **N** | **RR** | **95% CI** | **N** | **RR** | **95% CI** |
| --- | --- | --- | --- | --- | --- | --- |
|  |  |  |  |  |  |  |
|  |  |  |  |  |  |  |
| **Specific IgE** |  |  |  |  |  |  |
| House dust mite specific IgE >=0.35 kU_A_/L | 320 | **1.09** | **1.01-1.17** | 311 | **1.09** | **1.01-1.18** |
| Ryegrass specific IgE >=0.35 kU_A_/L | 320 | 1.09 | 0.98-1.21 | 311 | 1.05 | 0.94-1.17 |
| Alternaria specific IgE >=0.35 kU_A_/L | 319 | **1.18** | **1.07-1.30** | 310 | **1.14** | **1.03-1.27** |
| Cat dander specific IgE >=0.35 kU_A_/L | 320 | **1.22** | **1.02-1.46** | 311 | 1.15 | 0.95-1.38 |
|  |  |  |  |  |  |  |
| **Positive skin prick tests** |  |  |  |  |  |  |
| Any | 382 | **1.08** | **1.02-1.14** | 370 | 1.05 | 0.99-1.12 |
| Inhalant allergens | 382 | **1.08** | **1.02-1.15** | 370 | 1.06 | 1.00-1.13 |
| Ingested allergens | 382 | 1.16 | 1.00-1.34 | 370 | 1.09 | 0.91-1.32 |
| House dust mite | 382 | **1.08** | **1.00-1.16** | 370 | 1.06 | 0.98-1.15 |
| Ryegrass | 381 | **1.13** | **1.02-1.25** | 370 | 1.06 | 0.94-1.20 |
| Grass mix | 380 | **1.15** | **1.01-1.31** | 369 | 1.09 | 0.95-1.26 |
| Alternaria | 380 | 1.14 | 0.99-1.32 | 369 | 1.07 | 0.91-1.26 |
| Cockroach | 381 | 1.15 | 0.93-1.43 | 370 | 1.19 | 0.94-1.52 |
| Cat dander | 382 | 1.16 | 0.95-1.42 | 370 | 1.08 | 0.87-1.35 |
| Aspergillus | 381 | 0.92 | 0.59-1.43 | 370 | 0.90 | 0.53-1.55 |

RR is the Relative Risk per unit increase in weighted road density from Poisson regression with robust standard errors conducted on binary variables.

^ǂ^  Multivariate analyses are adjusted for sex, father’s education, mother’s education, environmental tobacco smoke exposure, breastfed to 6 months, any dog owned by 8 years, any cat owned by 8 years, maternal smoking in pregnancy, gas cooking at home.

## Table S3 Overall Sample in relation to weighted road density within 75m radius of home . Univariate and multivariate linear regression for spirometry and Poisson regression with robust standard error analyses for AHR, eNO, questionnaire outcomes (all) in relation to weighted road density within 75m radius of home

| **Variables** |  | **Unadjusted** | |  |  | **Adjusted ^ǂ^** | |  |
| --- | --- | --- | --- | --- | --- | --- | --- | --- |
|  | **N** | **Percent difference(B)** | **95% CI** | **p-value**  **interaction term**^ǂǂ^ | **N** | **Percent difference(B))** | **95% CI** | **p-value**  **interaction term**^ǂǂ^ |
| **Total IgE** |  |  |  |  |  |  |  |  |
| Total IgE (kU/L) † | **303** | **21.67** | **5.75-39.98** | - | **294** | **20.05** | **3.63-39.06** | - |
| **Spirometry** |  |  |  |  |  |  |  |  |
| FEV1 pre bronchodilator (L) | 397 | -0.24 | -1.02-0.55 | 0.115 | 392 | -0.16 | -0.97-0.65 | 0.207 |
| FEV1 post bronchodilator (L) | 393 | -0.08 | -0.78-0.63 | 0.0585 | 388 | -0.01 | -0.73-0.71 | 0.0936 |
| FVC pre bronchodilator (L) | 391 | -0.07 | -0.84-0.72 | 0.224 | 386 | 0.01 | -0.79-0.81 | 0.282 |
| FVC post bronchodilator (L) | 387 | -0.19 | -0.91-0.54 | 0.406 | 382 | -0.12 | -0.85-0.61 | 0.49 |
| FEV1/FVC ratio pre bronchodilator | 391 | -0.23 | -0.75-0.29 | 0.124 | 386 | -0.23 | -0.77-0.31 | 0.213 |
| FEV1/FVC ratio post bronchodilator | 387 | 0.05 | -0.33-0.44 | 0.064 | 382 | 0.04 | -0.36-0.43 | 0.0984 |
| pre Peak Expiratory Flow (PEF) | **268** | **-2.17** | **-3.68--0.63** | 0.155 | **265** | **-2.36** | **-3.95--0.75** | 0.2 |
| post Peak Expiratory Flow (PEF) | **264** | **-2.36** | **-3.84--0.86** | 0.304 | **261** | **-2.37** | **-3.93--0.79** | 0.376 |
| Pre forced expiratory flow at 50% vital capacity (FEF_50_) | 268 | -1.8 | -3.91-0.36 | 0.614 | 265 | -2.13 | -4.30-0.09 | 0.805 |
| Post forced expiratory flow at 50% vital capacity(FEF_50_) | **264** | **-2.34** | **-4.05--0.60** | 0.103 | **261** | **-2.21** | **-3.98--0.41** | 0.143 |
| Pre forced expiratory flow at mid-expiratory phase(FEF_25-75_) | **268** | **-2.16** | **-3.86--0.43** | 0.73 | **265** | **-2.37** | **-4.14--0.56** | 0.643 |
| Post forced expiratory flow at mid-expiratory phase(FEF_25-75_) | **264** | **-2.35** | **-3.86--0.81** | 0.775 | **261** | **-2.27** | **-3.84--0.67** | 0.716 |
|  |  |  |  |  |  |  |  |  |
| eNO ppb † | 376 | 1.02 | 0.92-1.13 | 0.692 | 364 | 1.04 | 0.93-1.16 | 0.666 |
| AHR (yes/no) | 332 | 1.03 | 0.88-1.21 | 0.0784 | 321 | 1.01 | 0.87-1.17 | **0.0383** |
|  |  |  |  |  |  |  |  |  |
| **Questionnaire outcomes** |  | **RR** |  |  |  | **RR** |  |  |
| Ever doctor-diagnosed asthma | 419 | 1.04 | 0.97-1.11 | 0.779 | 398 | 1.01 | 0.94-1.09 | 0.906 |
| Wheeze in last 12 months | 419 | 1.05 | 0.95-1.16 | 0.689 | 398 | 1.02 | 0.92-1.13 | 0.692 |
| Cough in last 12 months | 419 | 1.02 | 0.98-1.05 | 0.508 | 398 | 1.01 | 0.98-1.05 | 0.462 |
| Cough more than 4 times in last 12 months | 419 | 1.04 | 0.93-1.15 | 0.218 | 398 | 1.04 | 0.93-1.15 | 0.194 |
| Current asthma (wheeze + diagnosis/AHR) | 419 | 1.07 | 0.96-1.19 | 0.973 | 398 | 1.04 | 0.93-1.17 | 0.811 |
| Current asthma (wheeze + asthma diagnosis) | 419 | 1.08 | 0.97-1.20 | 0.962 | 398 | 1.05 | 0.94-1.17 | 0.845 |
| Poor asthma control | 419 | 1.11 | 0.93-1.32 | 0.392 | 398 | 1.13 | 0.97-1.31 | 0.232 |
| Ever doctor-diagnosed rhinitis | 419 | **1.16** | **1.07-1.26** | 0.69 | 398 | **1.13** | **1.03-1.24** | 0.64 |
| Current rhinitis symptoms | 417 | 1.07 | 0.98-1.18 | 0.208 | 397 | 1.07 | 0.97-1.17 | 0.342 |
| Ever doctor-diagnosed eczema | 419 | 1.05 | 0.99-1.12 | 0.5 | 398 | 1.05 | 0.98-1.11 | 0.283 |
| Current eczema | 405 | 1.07 | 0.94-1.22 | 0.74 | 392 | 1.02 | 0.89-1.16 | 0.482 |

Lung function variables are logged, therefore percent differences by B= 100(e^β^ – 1) are presented for each unit increase in weighted road density, where β is the regression coefficient from linear regression analyses for continuous variables

RR is the Relative Risk per unit increase in weighted road density from Poisson regression with robust standard errors conducted on binary variables.

^ǂ^  Multivariate analyses are adjusted for sex, father’s education, mother’s education, environmental tobacco smoke exposure, breastfed to 6 months, any dog owned by 8 years, any cat owned by 8 years, maternal smoking in pregnancy, gas cooking at home. Univariate and multivariate lung function analyses included also adjustment for age, height and weight.

^ǂǂ^ p-value from additional analysis where an interaction term of traffic road density and atopy was included in the model.

## Table S4 Atopics in relation to weighted road density within 50m radius of home. Univariate and multivariate linear regression for spirometry and Poisson regression with robust standard error analyses for AHR, eNO, questionnaire outcomes (all) in relation to weighted road density within 50m radius of home.

| **Variables** |  | **Unadjusted** | |  | **Adjusted ^ǂ^** | |
| --- | --- | --- | --- | --- | --- | --- |
|  | **N** | **Percent difference(B)** | **95% CI** | **N** | **Percent difference(B)** | **95% CI** |
| **Spirometry** |  |  |  |  |  |  |
| FEV1 pre bronchodilator (L) | 161 | -0.5 | -3.31-2.39 | 159 | -0.92 | -3.90-2.16 |
| FEV1 post bronchodilator (L) | 160 | 0.57 | -1.95-3.16 | 158 | 0.14 | -2.53-2.89 |
| FVC pre bronchodilator (L) | 157 | 0.44 | -2.27-3.23 | 155 | -0.43 | -3.29-2.52 |
| FVC post bronchodilator (L) | 156 | 0.33 | -2.22-2.94 | 154 | -0.2 | -2.87-2.54 |
| FEV1/FVC ratio pre bronchodilator | 157 | -0.76 | -2.61-1.13 | 155 | -0.48 | -2.44-1.52 |
| FEV1/FVC ratio post bronchodilator | 156 | 0.35 | -1.01-1.72 | 154 | 0.27 | -1.15-1.71 |
| pre Peak Expiratory Flow (PEF) | 109 | -2.67 | -8.50-3.53 | 108 | -3.79 | -10.21-3.09 |
| post Peak Expiratory Flow (PEF) | 104 | -3.1 | -8.85-3.01 | 103 | -4.54 | -10.82-2.19 |
| Pre forced expiratory flow at 50% vital capacity (FEF_50_) | 109 | -2.84 | -11.13-6.21 | 108 | -4.53 | -13.19-5.00 |
| Post forced expiratory flow at 50% vital capacity(FEF_50_) | 104 | -4.9 | -11.90-2.65 | 103 | -7.12 | -14.66-1.08 |
| Pre forced expiratory flow at mid-expiratory phase(FEF_25-75_) | 109 | -2.89 | -9.75-4.50 | 108 | -4.18 | -11.63-3.90 |
| Post forced expiratory flow at mid-expiratory phase(FEF_25-75_) | 104 | -3.64 | -9.56-2.67 | 103 | -4.91 | -11.37-2.02 |
|  |  |  |  |  |  |  |
| eNO ppb † | 161 | 0.99 | 0.86-1.15 | 154 | 1.03 | 0.88-1.21 |
| AHR (yes/no) | 133 | 0.68 | 0.40-1.15 | 127 | 0.71 | 0.39-1.30 |
|  |  |  |  |  |  |  |
| **Questionnaire outcomes** |  | **RR** |  |  | **RR** |  |
| Ever doctor-diagnosed asthma | 173 | **1.22** | **1.02-1.46** | 166 | **1.23** | **1.01-1.51** |
| Wheeze in last 12 months | 173 | **1.27** | **1.01-1.59** | 166 | **1.27** | **1.01-1.62** |
| Cough in last 12 months | 173 | 1.06 | 0.92-1.22 | 166 | 1.08 | 0.93-1.26 |
| Cough more than 4 times in last 12 months | 173 | 1.25 | 0.98-1.59 | 166 | **1.36** | **1.05-1.75** |
| Current asthma (wheeze + diagnosis/AHR) | 173 | 1.27 | 0.99-1.63 | 166 | 1.29 | 0.98-1.69 |
| Current asthma (wheeze + asthma diagnosis) | 173 | **1.28** | **1.00-1.65** | 166 | **1.31** | **1.00-1.72** |
| Poor asthma control | 173 | 1.29 | 0.86-1.92 | 166 | 1.39 | 0.96-2.02 |
| Ever doctor-diagnosed rhinitis | 173 | 1.25 | 0.96-1.63 | 166 | 1.29 | 0.93-1.79 |
| Current rhinitis symptoms | 171 | **1.27** | **1.03-1.58** | 165 | **1.26** | **1.01-1.59** |
| Ever doctor-diagnosed eczema | 173 | 1.13 | 0.97-1.33 | 166 | **1.23** | **1.03-1.46** |
| Current eczema | 172 | 1.28 | 0.93-1.76 | 165 | 1.4 | 0.97-2.00 |

Lung function variables are logged, therefore percent differences by B= 100(e^β^ – 1) are presented for each unit increase in weighted road density, where β is the regression coefficient from linear regression analyses for continuous variables

RR is the Relative Risk per unit increase in weighted road density from Poisson regression with robust standard errors conducted on binary variables.

^ǂ^  Multivariate analyses are adjusted for sex, father’s education, mother’s education, environmental tobacco smoke exposure, breastfed to 6 months, any dog owned by 8 years, any cat owned by 8 years, maternal smoking in pregnancy, gas cooking at home. Univariate and multivariate lung function analyses included also adjustment for age, height and weight.

## Table S5 Non –atopics in relation to weighted road density within 50m radius of home. Univariate and multivariate linear regression for spirometry and Poisson regression with robust standard error analyses for AHR, eNO, questionnaire outcomes (all) in relation to weighted road density

| **Variables** |  | **Unadjusted** | |  | **Adjusted ^ǂ^** | |
| --- | --- | --- | --- | --- | --- | --- |
|  | **N** | **Percent difference(B)** | **95% CI** | **N** | **Percent difference(B))** | **95% CI** |
| **Spirometry** |  |  |  |  |  |  |
| FEV1 pre bronchodilator (L) | 197 | -0.83 | -3.00-1.38 | 195 | -0.57 | -2.84-1.75 |
| FEV1 post bronchodilator (L) | 194 | -0.77 | -2.80-1.29 | 192 | -0.67 | -2.76-1.45 |
| FVC pre bronchodilator (L) | 195 | -0.38 | -2.60-1.90 | 193 | 0.06 | -2.27-2.44 |
| FVC post bronchodilator (L) | 192 | -0.77 | -2.87-1.39 | 190 | -0.61 | -2.78-1.60 |
| FEV1/FVC ratio pre bronchodilator | 195 | -0.75 | -2.27-0.80 | 193 | -0.9 | -2.51-0.74 |
| FEV1/FVC ratio post bronchodilator | 192 | -0.23 | -1.34-0.90 | 190 | -0.3 | -1.48-0.89 |
| pre Peak Expiratory Flow (PEF) | 128 | -0.18 | -4.97-4.86 | 127 | -1.64 | -6.84-3.85 |
| post Peak Expiratory Flow (PEF) | 129 | -0.53 | -5.16-4.32 | 128 | -1.21 | -6.22-4.08 |
| Pre forced expiratory flow at 50% vital capacity (FEF_50_) | 128 | 0.79 | -5.40-7.38 | 127 | -1.49 | -7.97-5.45 |
| Post forced expiratory flow at 50% vital capacity(FEF_50_) | 129 | -0.18 | -4.91-4.77 | 128 | -1.43 | -6.31-3.71 |
| Pre forced expiratory flow at mid-expiratory phase(FEF_25-75_) | 128 | 0.19 | -4.91-5.57 | 127 | -1.65 | -7.06-4.08 |
| Post forced expiratory flow at mid-expiratory phase(FEF_25-75_) | 129 | -0.52 | -5.15-4.34 | 128 | -1.17 | -6.08-3.99 |
|  |  | **RR** |  |  | **RR** |  |
| eNO ppb † | 202 | 0.99 | 0.87-1.14 | 197 | 1.03 | 0.89-1.18 |
| AHR (yes/no) | 185 | **1.71** | **1.07-2.74** | 180 | **1.75** | **1.11-2.76** |
|  |  |  |  |  |  |  |
| **Questionnaire outcomes** |  |  |  |  |  |  |
| Ever doctor-diagnosed asthma | 209 | 0.95 | 0.67-1.34 | 204 | 0.97 | 0.72-1.30 |
| Wheeze in last 12 months | 209 | 0.88 | 0.50-1.55 | 204 | 0.89 | 0.50-1.60 |
| Cough in last 12 months | 209 | 0.96 | 0.84-1.10 | 204 | 0.95 | 0.84-1.07 |
| Cough more than 4 times in last 12 months | 209 | 0.8 | 0.40-1.59 | 204 | 0.82 | 0.46-1.46 |
| Current asthma (wheeze + diagnosis/AHR) | 209 | 0.96 | 0.48-1.95 | 204 | 0.99 | 0.46-2.14 |
| Current asthma (wheeze + asthma diagnosis) | 209 | 0.98 | 0.48-2.01 | 204 | 1.01 | 0.48-2.14 |
| Poor asthma control | 209 | 1.17 | 0.35-3.91 | 204 | 1.44 | 0.47-4.46 |
| Ever doctor-diagnosed rhinitis | 209 | 1.29 | 0.83-2.02 | 204 | 1.27 | 0.81-2.00 |
| Current rhinitis symptoms | 209 | 0.73 | 0.40-1.33 | 204 | 0.76 | 0.42-1.38 |
| Ever doctor-diagnosed eczema | 209 | 1.04 | 0.81-1.34 | 204 | 1 | 0.78-1.29 |
| Current eczema | 207 | 0.77 | 0.28-2.16 | 202 | 0.81 | 0.28-2.33 |

Lung function variables are logged, therefore percent differences by B= 100(e^β^ – 1) are presented for each unit increase in weighted road density, where β is the regression coefficient from linear regression analyses for continuous variables

RR is the Relative Risk per unit increase in weighted road density from Poisson regression with robust standard errors conducted on binary variables.

^ǂ^  Multivariate analyses are adjusted for sex, father’s education, mother’s education, environmental tobacco smoke exposure, breastfed to 6 months, any dog owned by 8 years, any cat owned by 8 years, maternal smoking in pregnancy, gas cooking at home. Univariate and multivariate lung function analyses included also adjustment for age, height and weight.

## Table S6 Atopics in relation to weighted road density within 75m radius of home. Univariate and multivariate linear regression for spirometry and Poisson regression with robust standard error analyses for AHR, eNO, questionnaire outcomes (all) in relation to weighted road density within 75m radius of home

| **Variables** |  | **Unadjusted** | |  | **Adjusted ^ǂ^** | |
| --- | --- | --- | --- | --- | --- | --- |
|  | **N** | **Percent difference(B)** | **95% CI** | **B** | **Percent difference(B)** | **95% CI** |
| **Spirometry** |  |  |  |  |  |  |
| FEV1 pre bronchodilator (L) | 161 | 0.34 | -0.87-1.57 | 159 | 0.26 | -1.01-1.55 |
| FEV1 post bronchodilator (L) | 160 | 0.69 | -0.38-1.77 | 158 | 0.66 | -0.46-1.78 |
| FVC pre bronchodilator (L) | 157 | 0.57 | -0.59-1.75 | 155 | 0.48 | -0.74-1.71 |
| FVC post bronchodilator (L) | 156 | 0.36 | -0.73-1.46 | 154 | 0.36 | -0.77-1.49 |
| FEV1/FVC ratio pre bronchodilator | 157 | -0.18 | -0.98-0.63 | 155 | -0.28 | -1.11-0.55 |
| FEV1/FVC ratio post bronchodilator | 156 | 0.35 | -0.23-0.93 | 154 | 0.21 | -0.38-0.80 |
| pre Peak Expiratory Flow (PEF) | **109** | **-2.56** | **-4.90--0.17** | **108** | **-3.01** | **-5.52--0.43** |
| post Peak Expiratory Flow (PEF) | **104** | **-2.79** | **-5.10--0.42** | **103** | **-2.79** | **-5.30--0.21** |
| Pre forced expiratory flow at 50% vital capacity (FEF_50_) | **109** | **-3.31** | **-6.64-0.14** | **108** | **-4.71** | **-8.05--1.24** |
| Post forced expiratory flow at 50% vital capacity(FEF_50_) | **104** | **-3.52** | **-6.38--0.58** | **103** | **-3.83** | **-6.91--0.64** |
| Pre forced expiratory flow at mid-expiratory phase(FEF_25-75_) | **109** | **-3.14** | **-5.88--0.32** | **108** | **-3.95** | **-6.84--0.98** |
| Post forced expiratory flow at mid-expiratory phase(FEF_25-75_) | **104** | **-3.16** | **-5.54--0.73** | **103** | **-3.35** | **-5.91--0.72** |
|  |  | **RR** |  |  | **RR** |  |
| eNO ppb † | 161 | 1 | 0.94-1.06 | 154 | 1 | 0.93-1.06 |
| AHR (yes/no) | 133 | 0.91 | 0.72-1.15 | 127 | 0.91 | 0.72-1.15 |
|  |  |  |  |  |  |  |
| **Questionnaire outcomes** |  |  |  |  |  |  |
| Ever doctor-diagnosed asthma | 173 | 1.02 | 0.94-1.11 | 166 | 1.01 | 0.92-1.11 |
| Wheeze in last 12 months | 173 | 1.03 | 0.93-1.15 | 166 | 1.02 | 0.91-1.13 |
| Cough in last 12 months | 173 | 1.02 | 0.97-1.07 | 166 | 1.02 | 0.98-1.07 |
| Cough more than 4 times in last 12 months | 173 | 1.05 | 0.95-1.17 | 166 | 1.08 | 0.98-1.20 |
| Current asthma (wheeze + diagnosis/AHR) | 173 | 1.03 | 0.92-1.16 | 166 | 1.02 | 0.90-1.15 |
| Current asthma (wheeze + asthma diagnosis) | 173 | 1.04 | 0.93-1.17 | 166 | 1.03 | 0.91-1.16 |
| Poor asthma control | 173 | 1.04 | 0.86-1.25 | 166 | 1.04 | 0.86-1.25 |
| Ever doctor-diagnosed rhinitis | **173** | **1.13** | **1.03-1.23** | **166** | **1.13** | **1.01-1.25** |
| Current rhinitis symptoms | 171 | 1.08 | 0.99-1.17 | 165 | 1.05 | 0.97-1.15 |
| Ever doctor-diagnosed eczema | 173 | 1.05 | 0.99-1.11 | **166** | **1.07** | **1.01-1.14** |
| Current eczema | 172 | 1.03 | 0.89-1.19 | 165 | 1.05 | 0.92-1.21 |

Lung function variables are logged, therefore percent differences by B= 100(e^β^ – 1) are presented for each unit increase in weighted road density, where β is the regression coefficient from linear regression analyses for continuous variables

RR is the Relative Risk per unit increase in weighted road density from Poisson regression with robust standard errors conducted on binary variables.

^ǂ^  Multivariate analyses are adjusted for sex, father’s education, mother’s education, environmental tobacco smoke exposure, breastfed to 6 months, any dog owned by 8 years, any cat owned by 8 years, maternal smoking in pregnancy, gas cooking at home. Univariate and multivariate lung function analyses included also adjustment for age, height and weight.

## Table S7 Non-Atopics in relation to weighted road density within 75m radius of home. Univariate and multivariate linear regression for spirometry and Poisson regression with robust standard error analyses for AHR, eNO, questionnaire outcomes (all) in relation to weighted road density within 75m radius of home

| **Variables** |  | **Unadjusted** | |  | **Adjusted ^ǂ^** | |
| --- | --- | --- | --- | --- | --- | --- |
|  | **N** | **Percent difference(B)** | **95% CI** | **N** | **Percent difference(B)** | **95% CI** |
| **Spirometry** |  |  |  |  |  |  |
| FEV1 pre bronchodilator (L) | 197 | -0.98 | -2.03-0.08 | 195 | -0.89 | -2.00-0.24 |
| FEV1 post bronchodilator (L) | 194 | -0.8 | -1.79-0.19 | 192 | -0.78 | -1.81-0.26 |
| FVC pre bronchodilator (L) | 195 | -0.66 | -1.74-0.44 | 193 | -0.44 | -1.59-0.73 |
| FVC post bronchodilator (L) | 192 | -0.51 | -1.55-0.54 | 190 | -0.44 | -1.52-0.65 |
| FEV1/FVC ratio pre bronchodilator | 195 | -0.48 | -1.23-0.27 | 193 | -0.6 | -1.40-0.21 |
| FEV1/FVC ratio post bronchodilator | 192 | -0.42 | -0.96-0.13 | 190 | -0.48 | -1.06-0.11 |
| pre Peak Expiratory Flow (PEF) | 128 | -1.69 | -3.93-0.60 | 127 | -2.33 | -4.83-0.23 |
| post Peak Expiratory Flow (PEF) | 129 | -1.82 | -4.01-0.41 | 128 | -2.05 | -4.49-0.46 |
| Pre forced expiratory flow at 50% vital capacity (FEF_50_) | 128 | -1.39 | -4.29-1.60 | 127 | -2.2 | -5.34-1.05 |
| Post forced expiratory flow at 50% vital capacity(FEF_50_) | 129 | -1.28 | -3.53-1.02 | 128 | -1.57 | -3.98-0.90 |
| Pre forced expiratory flow at mid-expiratory phase(FEF_25-75_) | 128 | -1.72 | -4.10-0.71 | 127 | -2.34 | -4.95-0.33 |
| Post forced expiratory flow at mid-expiratory phase(FEF_25-75_) | 129 | -1.71 | -3.90-0.53 | 128 | -1.68 | -4.09-0.80 |
|  |  | **RR** |  |  | **RR** |  |
| eNO ppb † | 202 | 0.98 | 0.92-1.05 | 197 | 0.98 | 0.92-1.05 |
| AHR (yes/no) | 185 | 1.21 | 0.97-1.52 | 180 | **1.3** | **1.06-1.59** |
|  |  |  |  |  |  |  |
| **Questionnaire outcomes** |  |  |  |  |  |  |
| Ever doctor-diagnosed asthma | 209 | 1.05 | 0.91-1.20 | 204 | 1.03 | 0.92-1.15 |
| Wheeze in last 12 months | 209 | 0.96 | 0.69-1.34 | 204 | 0.95 | 0.64-1.40 |
| Cough in last 12 months | 209 | 0.99 | 0.94-1.06 | 204 | 0.98 | 0.93-1.03 |
| Cough more than 4 times in last 12 months | 209 | 0.85 | 0.61-1.18 | 204 | 0.83 | 0.62-1.11 |
| Current asthma (wheeze + diagnosis/AHR) | 209 | 1.04 | 0.74-1.46 | 204 | 1.1 | 0.77-1.55 |
| Current asthma (wheeze + asthma diagnosis) | 209 | 1.05 | 0.75-1.47 | 204 | 1.09 | 0.77-1.54 |
| Poor asthma control | 209 | 1.22 | 0.88-1.67 | 204 | 1.28 | 0.96-1.73 |
| Ever doctor-diagnosed rhinitis | 209 | 1.07 | 0.86-1.35 | 204 | 1.08 | 0.88-1.33 |
| Current rhinitis symptoms | 209 | 0.88 | 0.65-1.19 | 204 | 0.9 | 0.68-1.19 |
| Ever doctor-diagnosed eczema | 209 | 1.01 | 0.89-1.13 | 204 | 0.98 | 0.86-1.11 |
| Current eczema | 207 | 0.96 | 0.66-1.41 | 202 | 0.94 | 0.60-1.47 |

Lung function variables are logged, therefore percent differences by B= 100(e^β^ – 1) are presented for each unit increase in weighted road density, where β is the regression coefficient from linear regression analyses for continuous variables

RR is the Relative Risk per unit increase in weighted road density from Poisson regression with robust standard errors conducted on binary variables.

^ǂ^  Multivariate analyses are adjusted for sex, father’s education, mother’s education, environmental tobacco smoke exposure, breastfed to 6 months, any dog owned by 8 years, any cat owned by 8 years, maternal smoking in pregnancy, gas cooking at home. Univariate and multivariate lung function analyses included also adjustment for age, height and weight.
